# Improving protein tertiary structure prediction by deep learning and distance prediction in CASP14

**DOI:** 10.1002/prot.26186

**Published:** 2021-07-27

**Authors:** Jian Liu, Tianqi Wu, Zhiye Guo, Jie Hou, Jianlin Cheng

**Affiliations:** ^1^ Department of Electrical Engineering and Computer Science University of Missouri Columbia Missouri USA; ^2^ Department of Computer Science Saint Louis University St. Louis Missouri USA

**Keywords:** inter‐residue distance prediction, protein quality assessment, protein structure prediction

## Abstract

Substantial progresses in protein structure prediction have been made by utilizing deep‐learning and residue‐residue distance prediction since CASP13. Inspired by the advances, we improve our CASP14 MULTICOM protein structure prediction system by incorporating three new components: (a) a new deep learning‐based protein inter‐residue distance predictor to improve template‐free (ab initio) tertiary structure prediction, (b) an enhanced template‐based tertiary structure prediction method, and (c) distance‐based model quality assessment methods empowered by deep learning. In the 2020 CASP14 experiment, MULTICOM predictor was ranked seventh out of 146 predictors in tertiary structure prediction and ranked third out of 136 predictors in inter‐domain structure prediction. The results demonstrate that the template‐free modeling based on deep learning and residue‐residue distance prediction can predict the correct topology for almost all template‐based modeling targets and a majority of hard targets (template‐free targets or targets whose templates cannot be recognized), which is a significant improvement over the CASP13 MULTICOM predictor. Moreover, the template‐free modeling performs better than the template‐based modeling on not only hard targets but also the targets that have homologous templates. The performance of the template‐free modeling largely depends on the accuracy of distance prediction closely related to the quality of multiple sequence alignments. The structural model quality assessment works well on targets for which enough good models can be predicted, but it may perform poorly when only a few good models are predicted for a hard target and the distribution of model quality scores is highly skewed. MULTICOM is available at https://github.com/jianlin-cheng/MULTICOM_Human_CASP14/tree/CASP14_DeepRank3 and https://github.com/multicom-toolbox/multicom/tree/multicom_v2.0.

## 1 INTRODUCTION

Protein structure prediction is to computationally predict the three‐dimensional (3D) structure of a protein from its one‐dimensional (1D) amino acid sequence, which is much more efficient and cost‐effective than the gold‐standard experimental structure determination methods such as X‐ray crystallography, nuclear magnetic resonance (NMR) spectroscopy, and cryo‐electron microscopy (cryo‐EM). Computational structure prediction becomes more and more useful for elucidating protein structures as its accuracy improves.^1^ Two kinds of structure prediction methods have been developed: template‐based modeling and template‐free (ab initio) modeling. Template‐based modeling (TBM) methods first identify protein homologs with known structures for a target protein and then use them as templates to predict the target's structure.^2^, ^3^ A common approach of identifying homologous templates is based on Hidden Markov Models.^4^ When no significant known template structures are identified, template‐free modeling (FM) is the only viable approach to build structures from protein sequences. Traditional FM methods, such as Rosetta,^5^ attempt to build tertiary structure by assembling the mini‐structures of small sequence fragments into the conformation of the whole protein according to the guidance of statistical energy functions. Other FM tools such as CONFOLD^6^ use inter‐residue contact predictions as distance restraints to guide protein folding. In the 13th Critical Assessment of Protein Structure Prediction (CASP13), AlphaFold,^7^ a FM method based on deep learning distance prediction achieved the highest accuracy on both TBM targets and FM targets. Other top CASP13 tertiary structure prediction methods such as Zhang Group,^8^ MULTICOM,^9^ and RaptorX^10^ were also driven by deep learning and contact/distance predictions.

Inspired by the advances, our CASP14 MULTICOM system is equipped with a new deep‐learning based protein inter‐residue distance predictor (DeepDist^11^, ^12^) to generate accurate contact/distance predictions, which is used by DFOLD (https://github.com/jianlin-cheng/DFOLD) and trRosetta^13^ to construct template‐free structural models. Moreover, the template‐based prediction in MULTICOM is simplified and enhanced by removing redundant template‐identification tools and using deeper multiple sequence alignments (MSAs) in template search, while the template libraries and sequence databases are updated continuously. In addition, 11 new features calculated from predicted inter‐residue distance/contact maps are used to predict the quality of protein models in conjunction with other features in DeepRank^9^ to rank and select protein models. As a result of the improvements, MULTICOM was ranked seventh in tertiary structure prediction and third in inter‐domain structure prediction in CASP14.

In CASP14, AlphaFold2, an end‐to‐end attention‐based deep learning predictor achieved the unparalleled accuracy in predicting tertiary structures. Instead of predicting the residue‐residue distance from multiple sequence alignments first and then reconstructing tertiary structures from the distances, it directly predicts 3D structures from multiple sequence alignments, indicating a new direction of the end‐to‐end prediction of tertiary structures needs to be pursued in the future.

## 2 MATERIALS AND METHODS

### 2.1 Overview of the MULTICOM system

**FIGURE 1:**
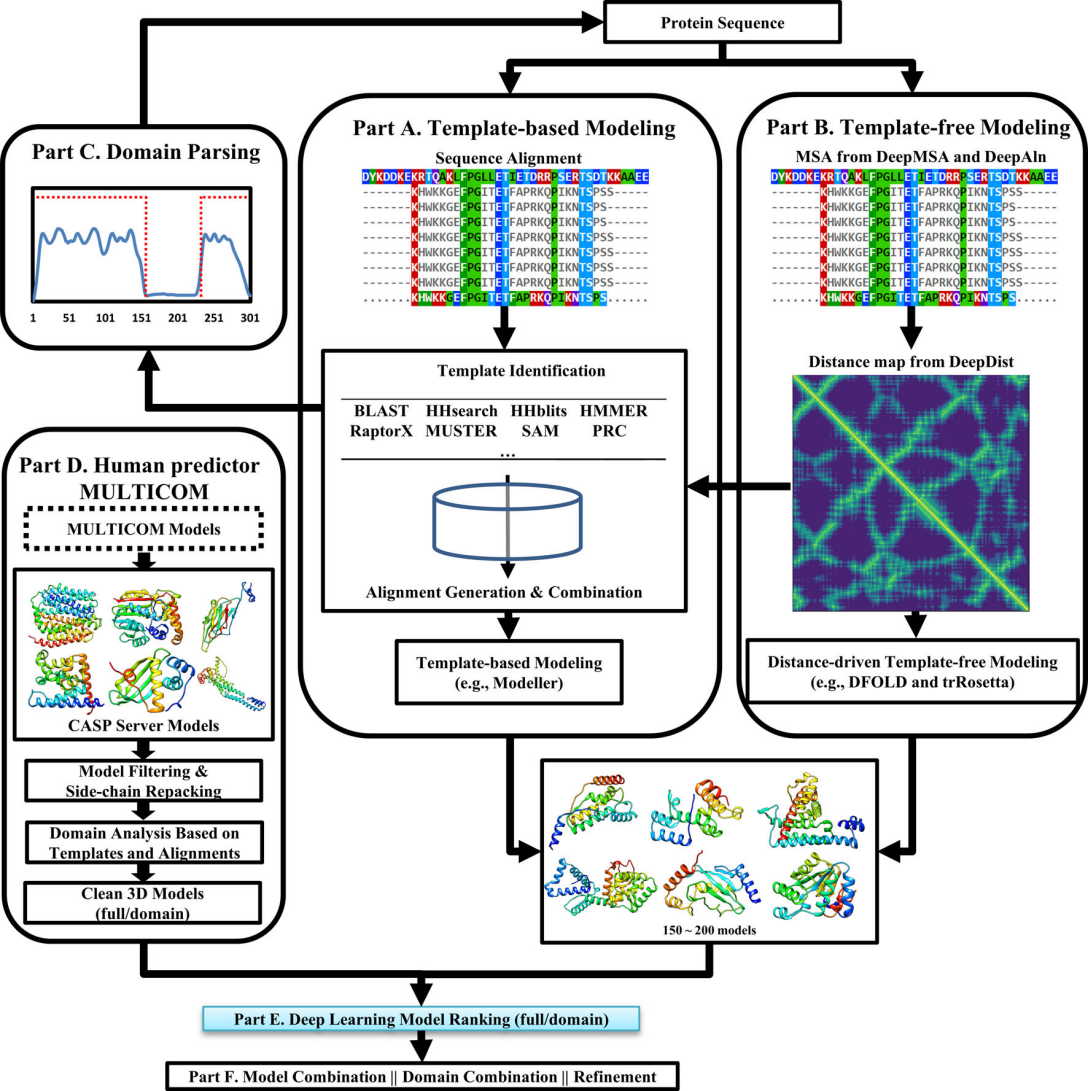
The pipeline of MULTICOM human and server protein structure predictors [Color figure can be viewed at wileyonlinelibrary.com]

The pipeline of MULTICOM human and automated server predictors can be roughly divided into six parts: template‐based modeling, template‐free modeling, domain parsing, model preprocessing, model ranking, and final model generation as depicted in Figure 1.

When a target protein sequence is received, the template‐based modeling (Figure 1, Part A) and template‐free modeling (Figure 1, Part B) start to run in parallel. In the template‐based modeling pipeline, MULTICOM first builds the multiple sequence alignments (MSA) for the target by searching it against sequence databases, which are used to generate sequence profiles. Then, the sequences profiles or the target sequence are searched against the template profile/sequence library by various alignment tools (BLAST,^14^ HHSearch,^15^ HHblits,^4^ HMMER,^16^ RaptorX,^17^ I‐TASSER/MUSTER,^18^, ^19^ SAM,^20^ PRC,^21^ and so on to identify templates and generate pairwise target‐template alignments. A combined target‐template alignment file is generated by combining the pairwise alignments. Structural models are built by feeding the combined alignment file into Modeller.^22^ In CASP14, the MULTICOM system was blindly tested as five automated servers. MULTICOM‐CLUSTER and MULTICOM‐CONSTRUCT servers used the template‐based prediction system described above, which was rather slow because it needed to run multiple sequence alignment tools. To speed up prediction, MULTICOM‐DEEP and MULTICOM‐HYBRID servers only used HHSearch and HHblits in the HHsuite package as well as PSI‐BLAST^23^ and HMMER to build sequence profiles and search for homologous templates, which are much faster than MULTICOM‐CLUSTER and MULTICOM‐CONSTRUCT. Considering that the distance‐based template‐free modeling can often achieve high accuracy on template‐based targets, we also tested MULTICOM‐DIST server predictor that completely skipped template‐based modeling and used only template‐free modeling for all the CASP14 targets.

In the newly developed distance‐based template‐free modeling pipeline (Figure 1, Part B), DeepMSA^24^ and DeepAln^11^ are used to generate two kinds of multiple sequence alignments, which are used to calculate residue‐residue coevolution features that are fed into different deep neural networks of DeepDist to predict the distance map—a two‐dimensional matrix representing the inter‐residue distances for the target protein. For some hard targets, the MSAs generated by HHblits on the Big Fantastic Database (BFD)^25^, ^26^ that contains hidden Markov model profiles of many proteins collected from metagenome sequence databases are also used to predict distance maps. The MSAs along with predicted distance maps are used to generate ab initio models with two different ab initio modeling tools (e.g., DFOLD and trRosetta^13^). In MULTICOM‐DEEP and MULTICOM‐HYBRID, distance maps and alignments generated by DeepMSA and DeepAln were also used to select templates for template‐based modeling. The detailed description of the distance‐guided template‐free modeling and its illustration (Figure S1) can be found in the Appendix S1.

Domain information (Figure 1, Part C) can be extracted from the target‐template sequence alignments. If no significant templates are found for a region of the sequence that is longer than 40 residues, the region is treated as a template‐free (FM) domain, otherwise a template‐based domain. The sequences of the domains are fed into the same pipeline above to build models for individual domains.

For the human predictor (Figure 1, Part D), all the CASP server models automatically downloaded from the CASP website and new models generated by MULTICOM servers if any are combined into one model pool as the initial input. Highly similar models from the same groups are filtered out if their pairwise global distance test score (GDT‐TS) score is greater than 0.95. SCWRL^27^ is used to repack the side chains for the models in the filtered model pool. If the target protein is predicted to have multiple domains, the full‐length models are split into domain models before model filtering.

Different quality assessment (QA) methods are used in MULTICOM to evaluate the models (Figure 1, Part E). In the server predictors, the models were assessed by APOLLO^28^ in MULTICOM‐CLUSTER and MULTICOM‐HYBRID, by DeepRank^9^ in MULTICOM‐CONSTRUCT, by SBROD^29^ in MULTICOM‐DIST, and by the average ranking score of APOLLO, SBROD and distance‐based rankings derived from distance map matching scores in MULTICOM‐DEEP. For the human prediction, two newly developed QAs (DeepRank3_Cluster and DeepRank_con) along with DeepRank used in CASP13 were used for model selection. DeepRank uses residue‐residue contacts predicted by DNCON2^30^ as input features, but DeepRank3_Cluster uses residue‐residue distances predicted by DeepDist as input features. DeepRank_con shares the same deep network with DeepRank but replaces contact predictions from DNCON2 with those from DeepDist. The three QAs also use other features including 1D structural features (e.g., predicted secondary structure, solvent accessibility) and the 3D model quality scores generated by different QA tools (e.g., RWplus,^31^ Voronota,^32^ Dope,^33^ and OPUS^34^).

Once the QAs generate the model rankings, final models are built by model combination, domain combination or model refinement (Figure 1, Part F) from top‐ranked models. For full‐length targets, top five ranked models are combined with other similar top‐ranked models (maximum 20 models) to generate the consensus models. If a target has multiple domains, top five models are generated by combining domain models using Modeller^22^ or AIDA.^35^ For the human prediction, if the combined models substantially deviate away from the original models, refinement tools (e.g., i3DRefine^36^ and ModRefiner^37^) will be used instead to refine the top‐ranked models to generate the final top five models for submission.

There are several additional differences between the human predictor and server predictors. First, the inputs for the human predictor are the server models from CASP including MULTICOM server models. Additional models generated by MULTICOM servers after the server submission deadline may be added into the model pool for some targets if any. Models filtering and side chain repacking are applied in the human prediction before feeding the models into the quality assessment methods. Second, in the human predictor, predicted domain boundaries are adjusted based on the top‐ranked models. Third, in the human prediction, the refinement tools are applied to improve the quality of top‐ranked models.

### 2.2 Protein model ranking

In the MULTICOM human predictor, three main quality assessment (QA) methods (DeepRank, DeepRank_con, and DeepRank3_Cluster) are applied to model selection. The methods share the similar features, including 1D features from predicted secondary structures and solvent accessibility and 3D QA scores from different QA tools (i.e., SBROD, RWplus,^31^ Voronota,^32^ Dope,^33^ and OPUS^34^, RF_CB_SRS_OD,^38^ DeepQA,^39^ ProQ2,^40^ ProQ3,^41^ APOLLO, Pcons^42^ and ModFOLDcluster2^43^), and differ mostly in 2D features derived from predicted contact or distance maps. DeepRank and DeepRank_con share the same neural network and are only different in the input contact map used to generate 2D features. In DeepRank, the input contact map is generated from DNCON2, but DeepRank_con takes an improved contact map from DeepDist as input. In DeepRank3_Cluster, the predicted distance map by DeepDist and the distance map calculated from a 3D model are used to calculate several distance map matching scores (i.e., SSIM & PSNR,^44^ GIST,^45^ RMSE, Recall, Precision, PHASH,^46^ Pearson correlation, and ORB^47^), which are combined with other 1D and 3D features as inputs. All the quality assessment methods apply the same two‐level network architecture. The first level of the network includes 10 neural networks trained by tenfold cross‐validation to predict the GDT‐TS scores of input models. Then the output scores are combined with initial input features to predict the final scores by the second level network. DeepRank, DeepRank_con and DeepRank3_Cluser were trained and tested on the models of the previous CASP experiments before they were blindly applied to the models of the CASP14 experiment.

### 2.3 Model refinement and combination

To improve the quality of selected top models, four different methods (e.g., model combination, i3DRefine, ModRefiner, and TM‐score based combination) are applied under different circumstances in the MULTICOM human predictor. After predicting the quality scores of the input server models, a standard protocol (Figure S2) is applied to generate the final top five models. Each top‐ranked model is combined with other top‐ranked models (maximum 20) that are similar to the start model (i.e., GDT‐TS > 0.6) to generate a consensus candidate model. If the GDT‐TS score between the consensus model and the start model is smaller than 0.9, the consensus model is discarded, and the candidate model is generated by using i3Drefine to refine the start model. ModRefiner is used alternatively if severe structural violations (e.g., atom clashes) exist in the candidate model or its secondary structures need to be further improved.

For some top models, if some of their good regions needed to be kept, but some bad regions needed to be replaced by the corresponding region in another model, a TM‐score based model combination method is applied. A superposed model is generated by aligning the two models using TMscore. A preliminary model is generated by replacing the bad region of the top model in the superimposed model with the corresponding region from the other model. The adjusted Ca atom trace of the top model is then extracted from the preliminary model to generate a combined model. The coordinates of other backbone atoms are added into the combined model using Pulchra.^48^ The side chains of the combined model are repacked by SCWRL according to the backbone structure. If needed, ModRefiner is applied to refine the model. This method can also be used to perform domain replacement.

### 2.4 Prediction of the structures of multidomain proteins

In the MULTICOM system, a domain detection algorithm based on the target‐template multiple sequence alignment generated by HHSearch or HHblits is applied to identify domains for multidomain proteins. Template sequences in the alignment are filtered out by their *E*‐value (>1), sequence length (≤40), or alignment coverage (≤0.5) for the target. If no template is left after filtering, the target is identified as a single‐domain template‐free target. Otherwise, further analysis is applied to the filtered alignment to identify domains. If a region of the target is not aligned with a template and has more than 40 residues, it is classified as a template‐free domain. All the other regions are classified as template‐based domains.

After splitting a multi‐domain target into domains, the sequence of each domain is fed to the prediction pipeline to generate structural models and the top five models for each domain are selected. Modeller is used by default to combine the top domain models into full‐length models. AIDA is used alternatively to combine domain models when the full‐length model generated by Modeller has severe clashes (i.e., the distance between any two Ca atoms is <1.9 Å) or broken chain (i.e., the distance between any two adjacent atoms is >4.5 Å). The domain‐based combination models may have good GDT‐TS scores for individual domains, but low scores when they are compared with the full‐length native structures because they do not have inter‐domain interaction information (e.g., relative position and orientation of domains). To address this problem, if a multidomain target does not have a significant template covering all its domains, domains are treated as independent modeling units and domain‐based combination models are used as top prediction. Otherwise, full‐length models generated without using domain information are selected based on the domain‐based model evaluation to maintain the domain–domain interactions. In some cases, both kinds of models are selected and added into the list of final top five predicted models.

## 3 RESULTS

In CASP14, both MULTICOM human and server predictors participated in the protein tertiary structure prediction. Among 92 CASP14 “all groups” domains for tertiary structure prediction, 54 domains are classified as template‐based (TBM‐easy or TBM‐hard) domains that have some structural templates in the Protein Data Bank (PDB) and 38 as FM or FM/TBM domains that have no templates or whose templates cannot be recognized. MULTICOM human predictor was ranked seventh among all the 146 predictors (see Table 1 for top 20 out 146 predictors and their total *Z*‐scores, average TM‐scores and average GDT‐TS scores) on 92 “all group” domains (https://predictioncenter.org/casp14/zscores_final.cgi) and third among all the 136 predictors (see Table 2 for the top 20 predictors' average and total *Z*‐scores) on 10 multidomain targets (e.g., T1030, T1038, T1052, T1053, T1058, T1061, T1085, T1086, T1094, and T1101) in the inter‐domain structure prediction category (https://predictioncenter.org/casp14/zscores_interdomain.cgi). After combining multiple server predictors from the same group as one entry, MULTICOM‐DEEP was ranked sixth after BAKER, RaptorX, Zhang, FEIG, and Seok groups on 58 (54 “all groups” +4 “server only”) TBM domains by the assessor's formula (Table S1). MULTICOM‐HYBRID server predictor was ranked fifth after Zhang, tFold, BAKER, and Yang groups on 38 FM or FM/TBM domains according to the assessor's formula (Table S2). The performance of our human and server prediction methods is systematically analyzed in the following sections using the official evaluation data downloaded from the CASP14's website.

**TABLE 1:** Top 20 predictors in CASP14 tertiary structure prediction ranked by *Z*‐score calculated from GDT‐TS

#	Group name	Sum *Z*‐score (>0.0)	Avg TM‐score	Avg GDT‐TS	#	Group name	Sum *Z*‐score (>0.0)	Avg TM‐score	Avg GDT‐TS
1	AlphaFold2	244.0217	0.9052	0.8801	11	tFold‐CaT_human	61.8464	0.6938	0.6229
2	BAKER	92.1241	0.7388	0.6695	12	FEIG‐R3	58.5809	0.6576	0.5942
3	BAKER‐experimental	91.4731	0.7334	0.6653	13	ropius0QA	57.8135	0.6891	0.6169
4	FEIG‐R2	74.5627	0.7088	0.6464	14	MUFOLD_H	55.9608	0.6659	0.6004
5	Zhang	68.8922	0.7142	0.6386	15	Zhang‐CEthreader	55.9467	0.6812	0.6064
6	tFold_human	65.2157	0.7021	0.6280	16	MESHI	55.9047	0.6861	0.6148
7	MULTICOM	64.0531	0.6989	0.6302	17	EMAP_CHAE	55.4235	0.6836	0.6129
8	QUARK	62.9711	0.6959	0.6234	18	BAKER‐ROSETTASERVER	55.2993	0.6511	0.5876
9	Zhang‐Server	62.9122	0.6978	0.6249	19	Wallner	55.1852	0.6760	0.6086
10	tFold‐IDT_human	62.0795	0.6862	0.6179	20	VoroMQA‐select	54.571	0.6814	0.6102

**TABLE 2:** Top 20 predictors in the inter‐domain structure prediction ranked by *Z*‐score based on F1 score + *Z*‐score based on Jaccard score + *Z*‐score based on best of contact agreement score

#	Group name	Sum *Z*‐score (>0.0)	Avg *Z*‐score (>0.0)	#	Group name	Sum *Z*‐score (>0.0)	Avg *Z*‐score (>0.0)
1	AlphaFold2	35.3062	3.5306	11	UOSHAN	7.2491	0.7249
2	BAKER‐experimental	15.717	1.4288	12	Ornate‐select	7.1811	0.6528
3	MULTICOM	8.986	0.8986	13	Bhattacharya	7.1549	0.7155
4	BAKER	8.759	0.8759	14	ProQ2	7.147	0.7147
5	ProQ3D	8.5411	0.8541	15	FEIG‐R1	6.8338	0.6834
6	FEIG‐R3	8.178	0.8178	16	NOVA	6.3867	0.6387
7	BAKER‐ROSETTASERVER	7.8402	0.784	17	Bilbul2020	6.3768	0.6377
8	EMAP_CHAE	7.8057	0.7806	18	RaptorX	6.3226	0.6323
9	VoroCNN‐select	7.5861	0.6896	19	DATE	6.3098	0.631
10	tFold‐CaT_human	7.532	0.7532	20	VoroMQA‐select	6.1055	0.6106

### 3.1 Performance of MULTICOM human predictor

Based on the official results on the CASP14 website, our MULTICOM human predictor was ranked seventh on all 92 domains overall, fourth on 54 TBM domains and 16th on 38 FM or FM/TBM domains in terms of the sum of the positive *Z*‐scores over the domains. The *Z*‐score of a model predicted for a target is the difference between the GDT‐TS score of the model and the average GDT‐TS score of all the models predicted for the target divided by the SD of the GDT‐TS scores of the models. A positive *Z*‐score indicates that the quality of the model is above the average model. The default CASP14 ranking uses the sum of positive *Z*‐scores over the domains to rank predictors in order not to penalize the new experimental methods that may predict bad models for some targets. Only seven human predictors from six different groups (AlphaFold2, BAKER, FEIG‐R2, Zhang, tFold_human, and MULTICOM) achieved higher performance than the best server predictor—QUARK (see Table 1). The average TM‐score of MULTICOM on the 92 “all‐group” domains is 0.6989, substantially higher than 0.5—a threshold for a correct fold prediction. If only the top one model per domain is considered, MULTICOM predicts the correct fold for 76 out of 92 (82.6%) domains (i.e., 98% TBM domains and 60.5% FM or FM/TBM domains). If the best of the top five models for each domain is considered, the success rate is increased to 84.8% (i.e., 98% TBM domains and 65.8% FM or FM/TBM domains).

**FIGURE 2:**
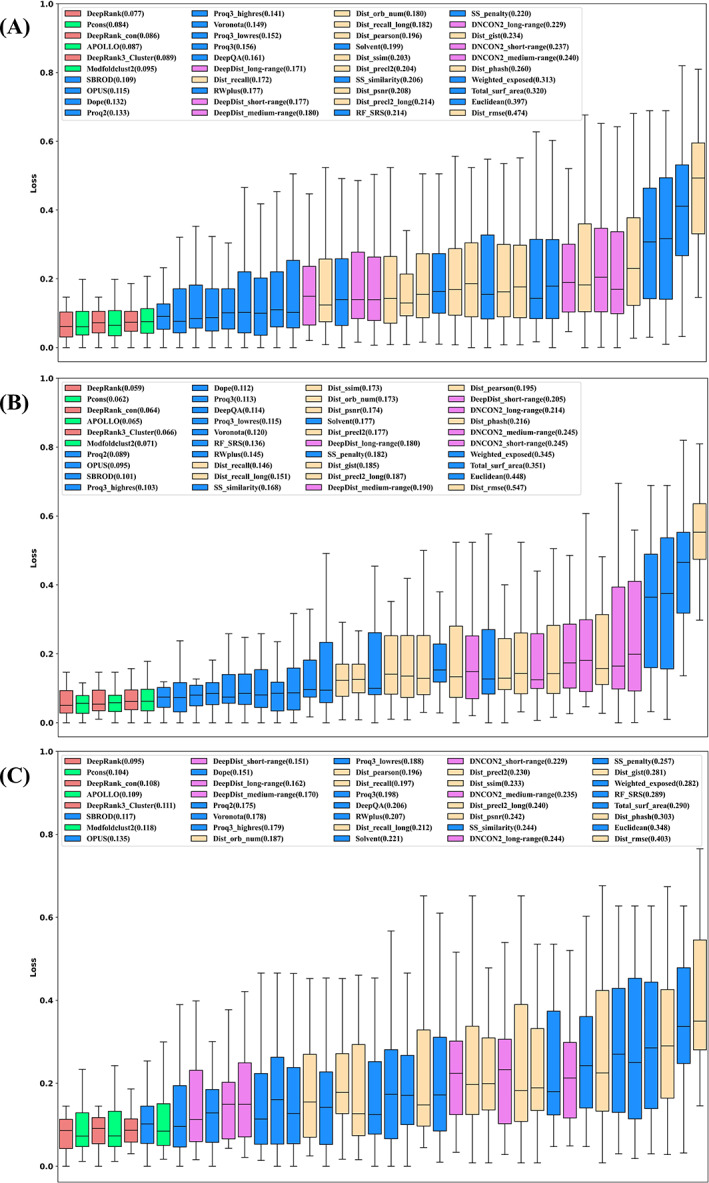
The average loss of 40 QA methods and features in MULTICOM. (A) the loss on 61 “all groups” full‐length targets. (B) the loss on 30 TBM‐easy or TBM‐hard full‐length targets. (C) the loss on 31 FM/TBM or FM full‐length targets. Red: three DeepRank methods including DeepRank, DeepRank_con, DeepRank3_Cluster; Green: three Multi‐model methods including APOLLO,^28^ Pcons,^42^ and ModFOLDcluster2^43^; Blue: 17 single‐model methods including (i.e., SBROD,^29^ RWplus,^31^ Voronota,^32^ Dope,^33^ OPUS_PSP,^34^ RF_CB_SRS_OD,^38^ DeepQA,^39^ ProQ2,^40^ ProQ3^41^); Pink: six contact matching scores including DeepDist/DNCON2 short‐range, medium‐range and long‐range contact matching scores; Yellow: 11 distance scores including SSIM and PSNR,^44^ GIST,^45^ RMSE, Recall, Precision, PHASH,^46^ Pearson correlation, and ORB^47^ [Color figure can be viewed at wileyonlinelibrary.com]

MULTICOM performed relatively better on TBM targets, but relatively worse on FM targets compared with some top predictors. The difference in the performance can be largely explained by the performance of the quality assessment (QA) methods. Figure 2(A) shows the ranking loss of all quality assessment methods or individual features, including three DeepRank method variants, three clustering‐based methods, six contact matching scores (long‐range, medium‐range, and short‐range matching scores), 11 distance scores, 17 single‐model methods used by MULTICOM on 61 “all groups” full‐length targets whose experimental structures have been released at CASP14 website. The loss of a method is the absolute difference between the true GDT‐TS scores of the best model for a target and the no. 1 model selected by a quality assessment method/feature. Figure 2(B,C) show the ranking loss on 30 TBM‐easy/TBM‐hard targets and 31 FM/TBM or FM targets. The difficulty of a multi‐domain target is classified as the most difficult category of its individual domains. According to the results, multi‐model QAs achieved the average performance similar to or better than the single‐model QAs on “all‐groups” targets, TBM targets and FM targets. Pcons and SBROD are the best multimodel QA and single‐model QA, respectively, according to the average loss of 61 “all‐groups” targets (Pcons: 0.084, SBROD: 0.109). Pcons had lower loss on 23 out of 30 TBM targets but higher loss on 16 out of 31 FM targets than SBROD, which demonstrates that multimodel and single‐model QA methods are complementary and both are valuable components in DeepRank. The average performance difference on 31 FM targets (Pcons: 0.104 vs. SBROD: 0.117) is smaller than on 30 TBM targets (Pcons: 0.062 vs. SBORD: 0.101). One reason is that multimodel QAs that depend on the structural comparison between models in the model pool usually perform better when the structural similarity between models in the pool is higher, which is the case for TBM targets, but single model QA methods that use the features extracted from a single model do not depend on pairwise model similarity and achieve more similar performance on TBM and FM targets. When the structural similarity between models is low, single‐model QAs are more likely to perform better than multimodel QAs than when the structural similarity between models is high. Despite the difference, both kinds of QAs have higher loss on hard (FM or FM/TBM) targets than on regular (TBM) targets, even though the performance of the multimodel QA method decreases more on hard targets, indicating that ranking models for hard targets is harder than for regular targets regardless of the type of QA methods. The reason is that the proportion of good models in the model pool of hard targets is generally lower than regular targets, which increases the difficulty of selecting good models. Among all the 40 QAs used, on average, DeepRank that combines single‐model and multimodel quality scores consistently outperformed the best multimodel QA (i.e., Pcons) and the best single‐model QA (i.e., SBROD) on 61 “all groups” targets (DeepRank: 0.077), 30 FM targets (DeepRank: 0.095) and 31 TBM targets (DeepRank: 0.059), respectively, proving the effectiveness of combining the complementary single‐model and multimodel quality scores for ranking protein models.

### 3.2 Performance of MULTICOM‐CLUSTER, MULTICOM‐CONSTRUCT, MULTICOM‐HYBRID, and MULTICOM‐DEEP server predictors using both template‐based and template‐free modeling

**FIGURE 3:**
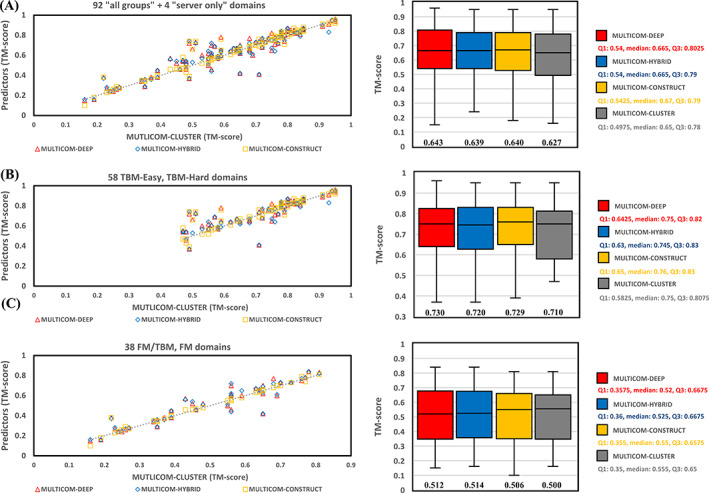
Evaluation of four MULTICOM server predictors in terms of the TM‐scores for the first submitted models. (A) On 92 “all group” +4 “server only” domains (left: TM‐scores of MULTICOM‐DEEP, MULTICOM‐HYBRID, MULTICOM‐CONSTRUCT models versus TM‐scores of MULTICOM‐CLUSTER models; right plot: mean and variation of the TM‐scores of the models of the four methods). (B) On 58 template‐based (TBM‐easy, TBM‐hard) domains. (C) On 38 FM or TBM/FM domains [Color figure can be viewed at wileyonlinelibrary.com]

**FIGURE 4:**
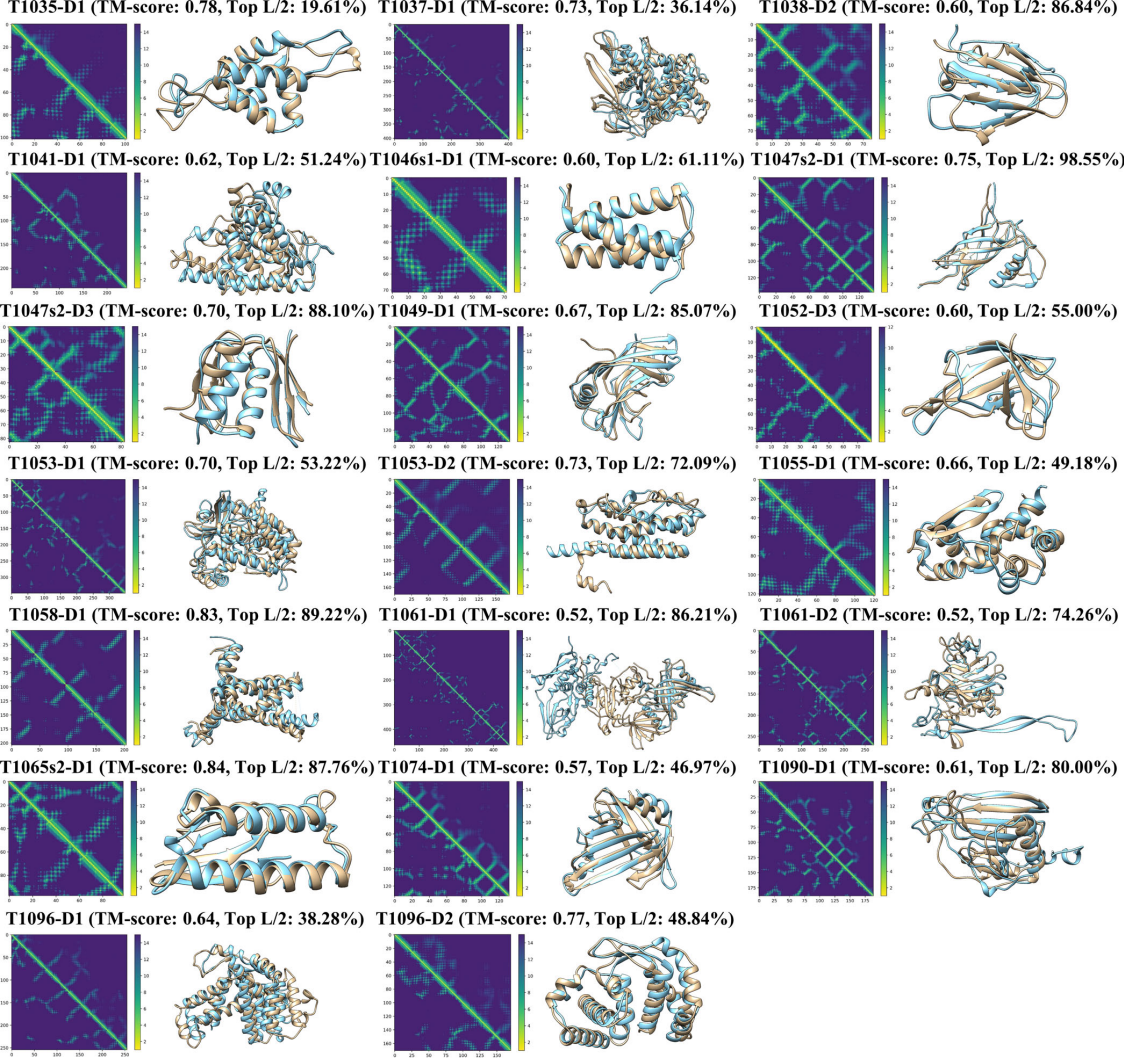
Predicted structures and distance maps compared with native structures and true distance maps for 20 FM or FM/TBM domains for which the first model predicted by MULTICOM‐DEEP has the correct topology (TM‐score > 0.5). For each domain, on the left is the comparison of the distance maps (lower triangle: true distance map; upper triangle: predicted distance map); and on the right is the comparison of predicted and true structures (light yellow: native structure, light blue: the first predicted structure). The TM‐score of the predicted structure and the precision of top *L*/2 long‐range contact predictions for each domain is listed on top of each sub‐figure [Color figure can be viewed at wileyonlinelibrary.com]

Figure 3 depicts the performance of the four server predictors on “all group” domains and “server only” domains, TBM domains, and FM or FM/TBM domains, respectively. For 92 “all group” and 4 “server only” domains, the average TM‐scores of the top‐1 models for these domains predicted by MULTICOM‐DEEP, MULTICOM‐HYBRID, MULTICOM‐CONSTRUCT, and MULTICOM‐CLUSTER are 0.643, 0.639, 0.640, and 0.627, respectively. The average TM‐scores of all the servers are substantially higher than 0.5, indicating the MULTICOM servers made good structure prediction for most domains on average. Specifically, if only the top‐1 model per domain is considered, MULTICOM‐DEEP predicts the correct topology for 75 out of 96 (78.1%) domains (i.e., 55 out of 58 (94.8%) TBM domains and 20 out of 38 (52.6%) FM or FM/TBM domains. Figure 4 illustrates the predicted structures and distance maps for the 20 FM or FM/TBM domains. If the best of five models for each domain is considered, the success rate is increased to 82.3% for all domains, 98.3% for TBM domains, and 57.9% for FM or FM/TBM domains. Overall, the performance of MULTICOM‐DEEP is significantly better than MULTICOM‐CONSTRUCT and MULTICOM‐CLUSTER (*P*‐values of one‐sided Wilcoxon signed rank test = .04724 and .00146, respectively), while MULTICOM‐HYBRID has the similar performance with MULTICOM‐DEEP (*P*‐value = .3042) and MULTICOM‐CONSTRUCT (*P*‐value = .1931) and the significantly better performance than MULTICOM‐CLUSTER (*P*‐value = .00437). On the 58 TBM domains, MULTICOM‐DEEP has the similar performance with MULTICOM‐HYBRID and MULTICOM‐CONSTRUCT (*P*‐values = .07044 and .1514, respectively). Their performances are significantly better than MULTICOM‐CLUSTER (*P*‐values = .00575, .03758, and .01492, respectively). Because the only difference between MULTICOM‐CLUSTER and MULTICOM‐CONSTRUCT lies in model quality assessment, the results show that DeepRank used by MULITCOM‐CONSTRUCT works better than APOLLO used by MULTICOM‐CLUSTER for ranking models of the TBM domains. The similar performance of MULTICOM‐DEEP, MULTICOM‐HYBRID, and MULTICOM‐CONSTRUCT on the TBM domains indicate that using HHSearch and HHblits to search for homologous templates in MULTICOM‐DEEP and MULTICOM‐HYBRID works at least as well as using many multiple alignment and threading tools in MULTICOM‐CONSTRUCT and MULTICOM‐CLUSTER while substantially reducing the search time. One reason is that HHSearch/HHblits is the most sensitive single tool for recognizing homologous templates as shown in the CASP13 experiment.^9^ Another reason is that the template‐free modeling can predict better models for many TBM domains than the template‐based modeling, which reduces the importance of the homologous template recognition for the final prediction.

Moreover, on the 38 FM or FM/TBM domains, MULTICOM‐HYBRID performed significantly better than MULTICOM‐CLUSTER (*P*‐value for one‐sided Wilcoxon signed rank test = .02499), while there is no significant difference between other servers (*P*‐values of one‐sided Wilcoxon signed rank test 0.05). Because MULTICOM‐HYBRID and MULTICOM‐CLUSTER used the same quality assessment method—APOLLO to select models, the results indicate that the quality of the model pool of MULTICOM‐HYBRID is better than MULTICOM‐CLUSTER. Indeed, replacing the distance maps predicted by trRosetta with the ones predicted by DeepDist can improve the performance of template‐free modeling. Furthermore, the average TM‐scores of all the four predictors on the FM or FM/TBM domains are ≥0.5, substantially better than the average 0.32 TM‐score of our CASP13 MULTICOM server predictors on the hard domains,^9^ indicating a substantial improvement on template‐free modeling has been made by our new template‐free structure prediction method.

### 3.3 Performance of the pure template‐free modeling server predictor MULTICOM‐DIST

**FIGURE 5:**
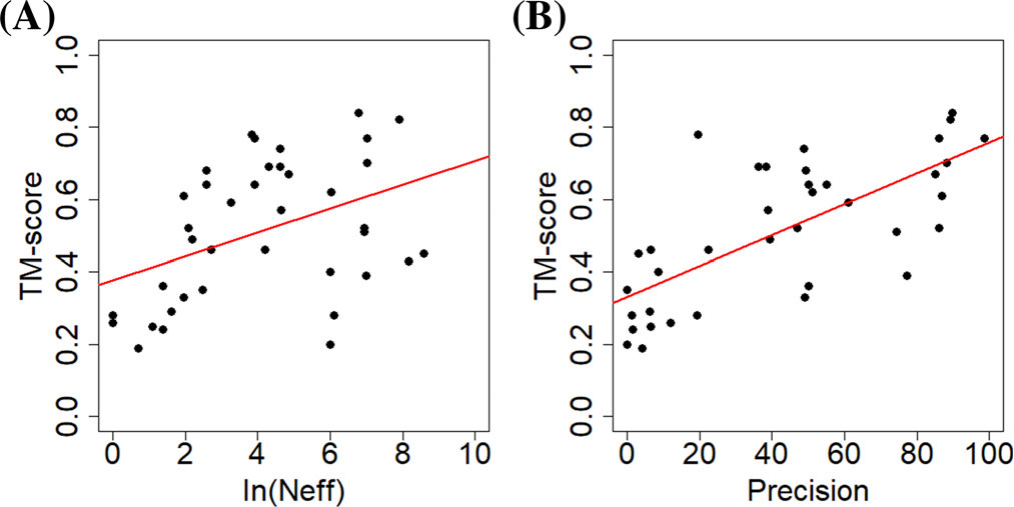
(A) Logarithm of Neff of MSAs versus the quality of MULTICOM‐DIST top‐1 models on the 38 CASP14 FM or FM/TBM domains. (B) The precision of top *L*/2 long‐range contact predictions versus the quality of MULTICOM‐DIST top‐1 models on the 38 FM or FM/TBM domains [Color figure can be viewed at wileyonlinelibrary.com]

The average TM‐score of top‐1 models predicted by MULTICOM‐DIST for the 38 CASP14 FM or FM/TBM domains is 0.513, which is similar to 0.514 of MULTICOM‐HYBRID or 0.512 of MULTICOM‐DEEP (*P*‐values of one‐sided Wilcoxon signed rank test = .5485 and .3976, respectively). The result is expected because they used a similar distance‐based template‐free modeling method. On the 38 CASP14 FM or FM/TBM domains, we investigate how different factors affect the model quality for the distance‐based template‐free modeling method. One is the number of effective sequences (Neff) in MSAs, measured as the number of the non‐redundant sequences at 62% sequence identity threshold. Figure 5(A) shows a weak correlation between the model quality and the logarithm of Neff (Pearson's correlation coefficient = 0.42) over all 38 domains. But when the logarithm of Neff is less than 6 (i.e., Neff <400), there is a strong correlation between the model quality and the logarithm of Neff (Pearson's correlation coefficient is 0.81). The results show the strong positive correlation between the model quality and Neff exists until Neff reaches about 400. Another factor investigated is the precision of distance prediction. Figure 5(B) shows a strong correlation between the precision of top *L*/2 contact prediction (*L*: sequence length) and the model quality (Pearson's correlation coefficient = 0.71), indicating that the model quality increases as the distance prediction gets more accurate.

On the 58 TBM domains, the average TM‐score of MULTICOM‐DIST based on template‐free modeling is 0.702, slightly less than 0.720 of MULTICOM‐HYBRID (*P*‐value = .2754 according to one‐sided Wilcoxon signed rank test) and significantly less than 0.730 of MULTICOM‐DEEP (*P*‐value = .03763) based on both template‐based and template‐free modeling. The results show that, even though integrating template‐based modeling and template‐free modeling may perform better than the pure template‐free modeling on some template‐based domains, the high TM‐score of MULTICOM‐DIST on the template‐based domains, which is close to that of MULTICOM‐HYBRID, demonstrates that the distance‐based template‐free modeling can work well on template‐based targets, which is consistent with the finding of AlphaFold in the CASP13 experiment. In fact, if only the top‐1 model is considered for each domain, MULTICOM‐DIST predicts the correct fold for 53 out of 58 (91.4%) TBM domains. If the best of five models is considered for each domain, the success rate is increased to 96.6%. The results confirm that the distance‐based protein structure prediction can universally address the protein structure prediction problem. Therefore, the traditional division of protein structure prediction into template‐based and template‐free modeling may not be necessary anymore, even though template‐based structural information can still be used in the modeling process.

**FIGURE 6:**
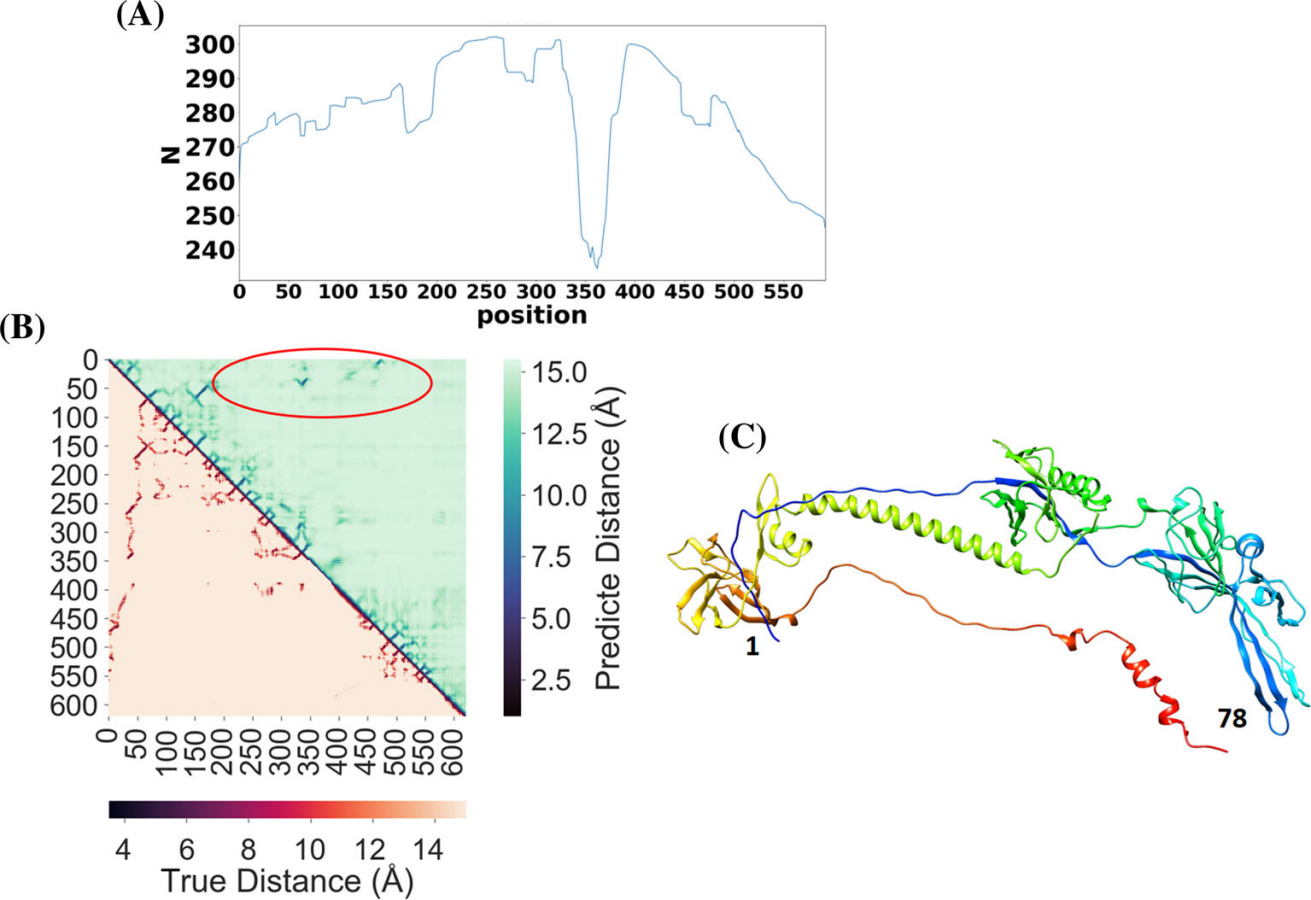
(A) The plot of the number of non‐gap residues of multiple sequence alignment of T1036s1 against residue positions, where *x*‐axis stands for each residue position and *y*‐axis stands for the number of non‐gap amino acids. (B) The true distance map of T1036s1‐D1 (lower triangle) versus the predicted distance map from MULTICOM‐DIST (upper triangle). (C) The true structure of target T1036s1‐D1 in rainbow, starting from the N‐terminal in blue to C‐terminal in red [Color figure can be viewed at wileyonlinelibrary.com]

The slightly worse average TM‐score of MULTICOM‐DIST on the template‐based domains was largely due to the lack of good treatment of large multi‐domain targets in the early stage of CASP14 experiment. For large proteins with sequence length >500, it was often hard to find enough well‐aligned homologous sequences covering the entire sequence for accurate full‐length residue–residue distance prediction. The global multiple sequence alignment could be dominated by one or two regions with a lot of homologous sequences, leaving the remaining regions not well aligned (i.e., many gaps). For one large TBM‐easy target T1036s1 of 818 residues long, MULTICOM‐DIST failed to construct the full‐length model for this target and its model had a very low TM‐score—0.19 for the domain T1036s1‐D1 (sequence region: 1–621). The number of effective sequences of the multiple sequence alignment for the target was 45 and the number of sequences in the multiple sequence alignment was 265, which were relatively small for the distance prediction for the entire target. For each residue position in the multiple sequence alignment of the target, we calculate the number of non‐gap amino acids in the position shown in Figure 6(A). There are few homologous sequences that can cover the entire sequence length. Most homologous sequences in the alignment only cover some regions of the target. There are many gaps in the region ranging from residue 300 to 400. Figure 6(B) compares the true distance map (lower triangle) and the predicted distance map (upper triangle). Even though the predicted distance map contains good intra‐domain distance predictions that are similar to the true distances, it does not have good long‐range inter‐domain distance predictions. The region inside the red circle in the predicted distance map denotes the place where long‐range inter‐residue contacts were not well predicted in comparison with the true distance map. The true contacts in the region correspond to the interactions between residues 1–78 and residues 57–551 (Figure 6(C)). Different from MULTICOM‐DIST, the other four MULTICOM server predictors found strong full‐length templates and constructed high‐quality models from the templates. For instance, MULTICOM‐CONSTRUCT found a significant template 3NWA with the sequence identity of 0.488, sequence coverage of 0.966, and *E*‐value of 5.7E‐226 and built a good model with TM‐score of 0.92. This example shows that more care needs to be taken for large multidomain proteins in template‐free modeling and it is useful to incorporate some template‐based distance information into the distance‐based free modeling.

### 3.4 Improved performance enabled by model combination

**FIGURE 7:**
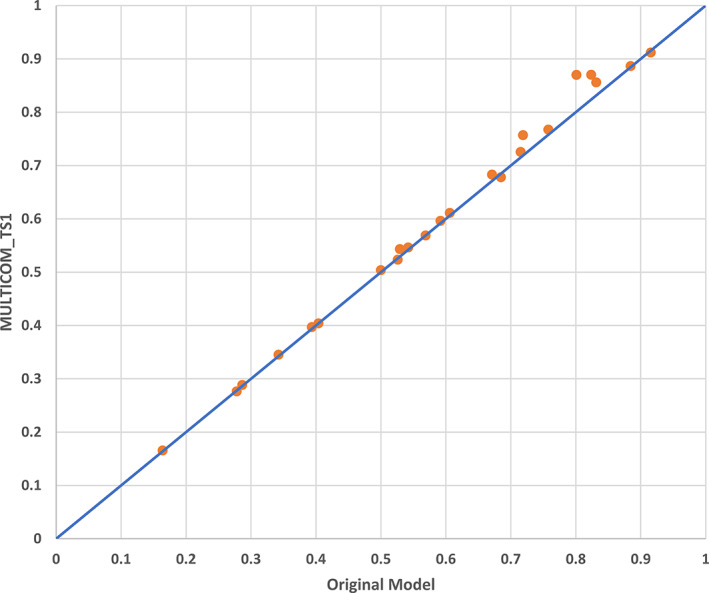
The GDT‐TS scores of original models versus the GDT‐TS scores of combined models (MULTICOM_TS1) [Color figure can be viewed at wileyonlinelibrary.com]

For the top‐ranked model (called the reference model or the original model) of some targets, the human predictor—MULTICOM—collected other models ranked within top 60 and having GDT‐TS larger than 0.7 or root mean square distance (RMSD) smaller than 3 Å with respect to the reference model. The reference model and the other selected models were used as templates for Modeller to build a combined model as the final prediction. During CASP14, this model combination approach was applied to 23 targets (Table S3). In Figure 7, the GDT‐TS scores of the original top‐1 models are plotted against the scores of the final, combined top‐1 model submitted to CASP14 (i.e., MULTICOM_TS1). Out of 23 targets, the quality of the combined models is better than the original models on 17 targets (73.9%), while on the rest of the targets their quality is only marginally worse (GDT‐TS difference <0.007). The *P*‐value of the one‐sided Wilcoxon signed rank test is .00125, showing that the model combination significantly improves model quality.

**FIGURE 8:**
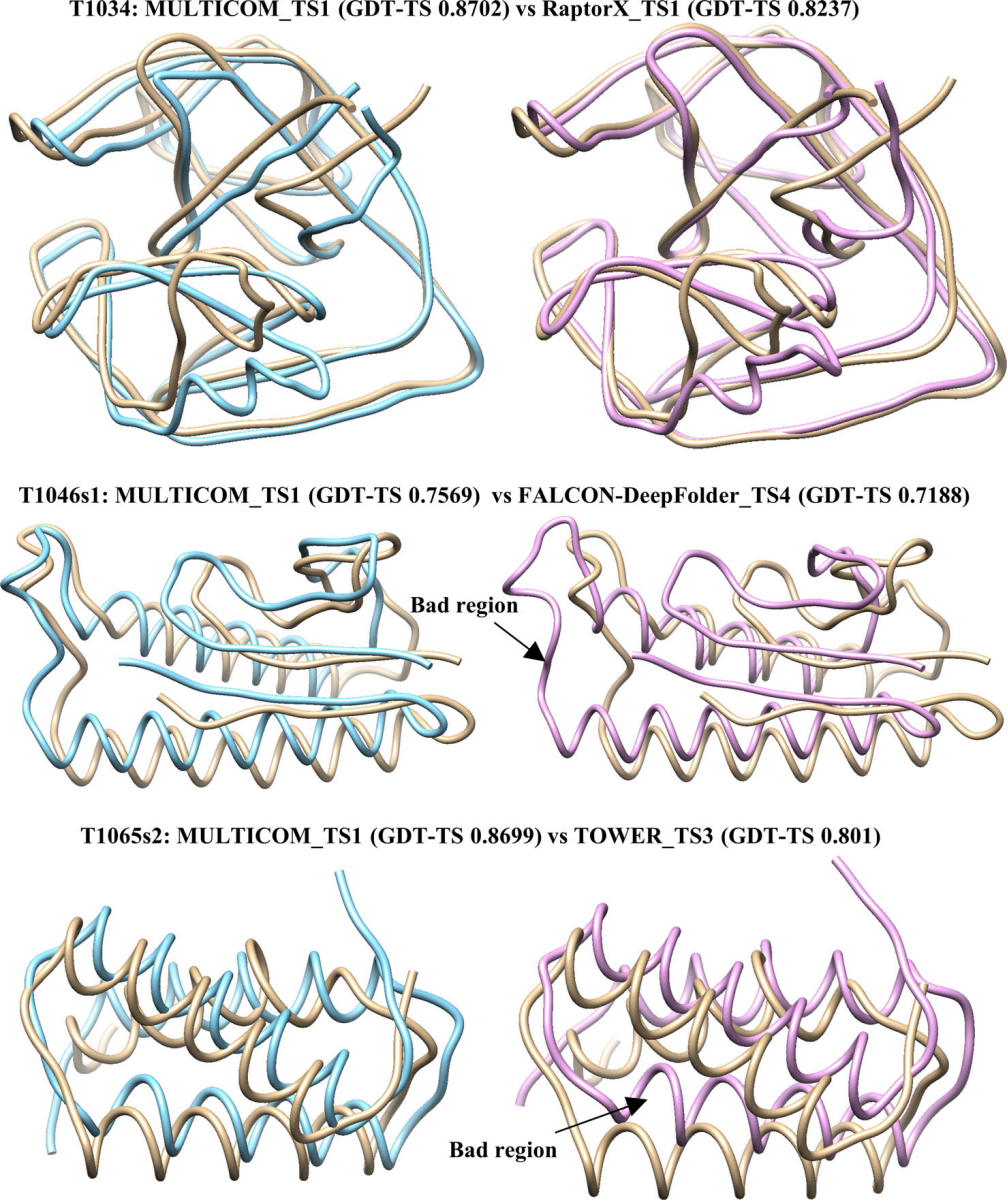
Good examples for model combination on targets T1034, T1046s1, and T1065s2 (light yellow: native structure, light blue: MULTICOM_TS1 [final combined model], pink: original model) [Color figure can be viewed at wileyonlinelibrary.com]

The performance of the model combination depends on the reference model as well as the number of the models combined. The Pearson correlation between the number of models combined and the GDT‐TS score difference (improvement) is 0.476, which indicates that including more templates tends to improve the performance. Figure 8 illustrates some good examples (i.e., T1034, T1046s1, and T1065s2). The combined model has smaller deviations from the true structure compared with the original models, whereas some poorly modeled region in the original model is fixed in the combined model for some targets (e.g., T1046s1).

There are some limitations in the model combination. For some FM targets (e.g., T1026, T1037, T1040, T1043, T1074, and T1086), few similar models can be found to combine with the original model, leading to very little or no improvement. Although the model combination method could improve the quality of the original model, the quality of the combined model may be still worse than the best model in the model pool if the quality of the original model is much worse than the best model.

### 3.5 Good and bad prediction examples

Among all 92 “all group” domains, MULTICOM human predictions were ranked in the top five for three domains in terms of the top‐1 model: T1034‐D1, T1092‐D1, and T1093‐D2. For T1034‐D1 (Figure S3), MULTICOM's model quality assessment selected RaptorX_TS1 as a start model, whose GDT‐TS is 0.8237. MULTICOM combined it with 19 other top‐ranked server models that were similar to the start model (i.e., GDT‐TS >0.6) to generate a final model. The GDT‐TS of the final top1 model (MULTICOM_TS1) is 0.8702, which is higher than the start model and is ranked only after the AlphaFold2 model. For T1092‐D1 and T1093‐D2, the full‐length protein sequences were divided into domains whose boundaries were close to the true domain definition. Based on the domain splitting, MULTICOM was able to select the best domain model in the server model pool as start models to generate high‐quality final models.

**FIGURE 9:**
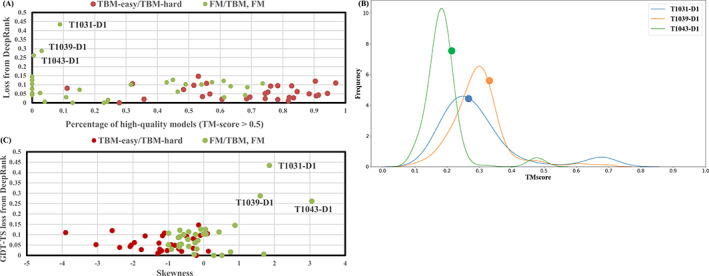
(A) The percentage of good‐quality models (TM‐score > 0.5) versus GDT‐TS loss of DeepRank. (B) The distribution of TM‐scores of the models of T1031‐D1 (green), T1039‐D1 (red), and T1043‐D1 (blue); dots on the curves denote the top model selected for the targets. (C) The skewness of TM‐scores of the models versus GDT‐TS losses of DeepRank for all 61 targets [Color figure can be viewed at wileyonlinelibrary.com]

MULTICOM performed relatively poorly on some FM/TBM or FM domains, including single‐domain targets: T1031‐D1, T1039‐D1, T1043‐D1, T1061‐D1. For T1031‐D1, T1039‐D1, and T1043‐D1. MULTICOM's quality assessment failed to select good start models from the model pool. One reason causing the failure is the number of good‐quality models in the model pool is low and the distribution of TM‐scores of the models for these targets is highly skewed. In Figure 9(A), the percentage of good‐quality models (TM‐score >0.5) is plotted against the GDT‐TS loss of the best quality assessment method—DeepRank. It is shown that TBM targets have a larger proportion of good‐quality models than FM or FM/TBM targets. Among five hard targets that have greater than 0% but less than 10% of good models, three of them (T1031‐D1, T1039‐D1, and T1043‐D1) have the highest loss among all the targets (>0.25). All the other targets have the loss less than 0.15, even for the targets that have no good models predicted at all (i.e., 0% good models). Figure 9(B) is the plot of the distribution of TM‐scores of the models for these three targets. DeepRank selected a model with the score close to the mode (the high‐density area) of the distribution instead of a good model in the extremely low‐density area. To further investigate how the distribution of the quality scores of the models in the model pool affects the performance of DeepRank, the skewness of the distribution is calculated for the targets and plotted against the loss on them (Figure 9(C)). The three targets with the highest loss have the highest skewness (i.e., 1.85 for T1031‐D1, 1.6 for T1039‐D1, and 3.05 for T1043‐D1), where the positive (negative) value of skewness indicates that the mean TM‐score is larger (less) than the median TM‐score. On 31 FM or FM/TBM targets, the correlation between the skewness and the loss of DeepRank is 0.56, lower than 0.71 of Pcons, indicating that both methods are affected by the skewness, but DeepRank integrating both multimodel and single model features is more robust against the skewness than a clustering‐based multimodel method. Another reason for the ranking failure is the incorrect domain prediction. For T1061, a long 949‐residue long target, MULTICOM failed to detect the correct domain boundaries, which led to the bad prediction for its first domain (T1061‐D1). The example demonstrates that the accuracy of domain prediction has a significant impact on the tertiary structure prediction for some multidomain targets.

## 4 DISCUSSION

It has been known that the template‐free (ab initio) modeling generally works much better than the template‐based modeling on FM targets that have no templates. To confirm this, we compared the quality of top‐1 template‐based models predicted by MULTICOM‐HYBRID and top‐1 template‐free models predicted by MULTICOM‐DIST for 33 FM or FM/TBM domains (Table S4). Five FM or FM/TBM domains—T1052‐D3, T1061‐D1, T1061‐D2, T1080‐D1, and T1085‐D2—were excluded from this analysis due to lack of true structures or predicted full‐length template‐based or template‐free models. Indeed, the template‐free modeling of MULTICOM‐DIST performs better than the template‐based modeling of MULTICOM‐HYBRID on 32 of 33 domains (i.e., 97%), and the average TM‐score of the template‐free models is more than double that of the template‐based models (i.e., 0.513 of the template‐free modeling versus 0.211 of the template‐based modeling).

However, it is less clear if template‐free modeling can beat template‐based modeling on TBM targets for which some homologous templates can be found. CASP13 experiment showed that template‐free modeling^7^, ^8^, ^49^ worked better than template‐based modeling on quite some TBM targets. To further investigate this, we compared the quality of template‐based models predicted by MULTICOM‐HYBRID and template‐free models predicted by MULTICOM‐DIST on 47 out of 58 CASP14 TBM domains (see Table S5), while the other 11 domains (T1052‐D1, T1052‐D2, T1061‐D3, T1091‐D1, T1091‐D2, T1091‐D3, T1091‐D4, T1085‐D1, T1085‐D3, T1086‐D1, and T1086‐D2) were excluded from this analysis due to lack of native structures or predicted full‐length template‐based or template‐free models. The average TM‐score of the models generated by the template‐based modeling of MULTICOM‐HYBRID for the TBM domains is 0.636, which is significantly lower than 0.703 (*P*‐value = .01724 according to one‐sided Wilcoxon signed rank test) of the template‐free modeling of MULTIOM‐DIST. The template‐free modeling performs better than the template‐based modeling on 59.6% of TBM domains (28 out of 47).

An interesting observation is that the performance of the template‐free modeling on the 33 FM or FM/TBM domains (MULTICOM‐DIST's average TM‐score on them = 0.513) is significantly worse than on the 47 TBM domains (MULTICOM‐DIST's average TM‐score on them = 0.703) (*P*‐value for one‐sided Wilcoxon rank sum test <.00005). The Pearson correlation between the TM‐scores of the top‐1 models and the precision of long‐range top *L*/2 contact predictions on the 80 (47 TBM + 33 FM or FM/TBM) domains is 0.72, showing that the precision of the contact/distance predictions made by DeepDist largely determines the quality of the structural models built by the distance‐based template‐free modeling. The average precision of top *L*/2 contact predictions for the TBM domains is 69.16%, which is significantly higher than 41.45% for the FM or FM/TBM domains (*P*‐value <.0001 according to one‐sided Wilcoxon rank test). Therefore, the better tertiary structure prediction performance of the template‐free modeling on the TBM domains is largely due to the higher accuracy of contact/distance prediction on them.

We conduct further analyses to investigate the two possible reasons that may cause the higher accuracy of contact/distance prediction and tertiary structure prediction for the TBM domains: (a) the better quality of MSAs of the TBM domains and (b) the existence of homologs in the training data of DeepDist for the TBM domains. Firstly, we calculate the Neff of the MSAs to approximately measure their quality. The average Neff of MSAs for the TBM domains is 2557, which is significantly larger than 286 for the FM and FM/TBM domains (*P*‐value <.00001 according to Wilcoxon rank sum test). The Pearson's correlation between the logarithm of Neff and the precision of top *L*/2 long‐range contact predictions is 0.616 and between the logarithm of Neff and the TM‐score of top‐1 models is 0.623, indicating the higher quality of MSAs is one important reason causing the higher accuracy of tertiary structure prediction and contact/distance prediction for the TBM domains. The result is expected because better MSAs leads to more reliable co‐evolutionary input features for DeepDist to make better distance predictions and more accurate distance predictions lead to better tertiary structural models reconstructed from them.

Secondly, to investigate if the existence of homologs in the training dataset of DeepDist for the TBM domains may also contribute to the higher accuracy of contact/distance predictions and tertiary structure prediction, we collect all 10 pairs of TBM and FM (or FM/TBM) domains whose MSAs have the same or very similar Neff (i.e., the difference of Neff ≤5%) to compare. The 10 TBM domains form one set (TBM_Set) and their FM (or FM/TBM) counterparts form another set (FM_Set). Because the domains from the two sets have very similar alignment quality in terms of Neff, the main difference between them is that the domains in TBM_Set tend to have homologs in the training dataset of DeepDist, but the domains in FM_Set do not. The average precision of the long‐range contact predictions for TBM_Set is 50.40%, which is higher than 48.02% for FM_Set, but the difference is not significant (*P*‐value = .4192 according to the Wilcoxon signed rank test). Similarly, the average TM‐score of the top‐1 models for TBM_Set is 0.607, which is higher than 0.5514 for FM_Set, but the difference is not significant (*P*‐value = .1795 according to the Wilcoxon signed rank test). The comparison on these two relatively small datasets (i.e., the sample size of each set = 10) seems to suggest that the existence of homologs for the TBM domains in the DeepDist's training dataset may make some insignificant contribution to the increase in the prediction accuracy.

The two analyses above together indicate that the accuracy of the template‐free structure prediction and contact/distance prediction for a target is largely influenced by the quality of its multiple sequence alignment, and to a less extent, may also be affected by whether it has some homology with the proteins used to train the distance predictor.

Although it was not clear to us that our template‐free modeling would work better than the template‐based modeling on both TBM and FM targets prior to the CASP14 experiment, it is interesting to see MULTICOM‐HYBRID selected a template‐free model as top‐1 model for most CASP14 (TBM, FM, and FM/TBM) targets. It sometimes selected template‐based models as top‐1 model only when a very significant template was found for a target (e.g., the *e*‐value of the best template hit < *E*‐20). Generally, MULTICOM‐HYBRID prefers FM models over TBM models regardless of the type of the targets.

## 5 CONCLUSION

We developed the MULTICOM protein structure prediction system for the CASP14 experiment and evaluated and analyzed its performance on the CASP14 targets. We demonstrate that the distance‐based template‐free prediction empowered by deep learning significantly improves the accuracy of protein tertiary structure prediction. The approach can work well on both template‐free and template‐based targets and therefore can be applied to elucidate the structures of many proteins without known structures in a genome. However, the quality of template‐free modeling critically depends on the quality of deep learning‐based residue–residue distance prediction, which in turns depends on the quality of multiple sequence alignment. In contrast to the substantial improvement in template‐free structure prediction, there is little improvement in protein model quality assessment in our CAS14 system over the CASP13 methods. The quality assessment methods using more accurate residue–residue distance prediction features did not perform better than the quality assessment method using only residue–residue contact prediction features, suggesting that better methods of using distance predictions in quality assessment are needed. Moreover, domain prediction plays an important role in both model generation and evaluation. Accurate domain prediction can help generate better tertiary structure models and select better predicted models for some multidomain targets.

### PEER REVIEW

The peer review history for this article is available at https://publons.com/publon/10.1002/prot.26186.

## Supporting information


**Appendix S1**: Supporting Information

## Data Availability

Data sharing is not applicable to this article as no new data were created or analyzed in this study.
