# Supplementary material for: Improving protein tertiary structure prediction by deep learning and distance prediction in CASP14
Source: Proteins. 2021 Jul 27;90(1):58–72. doi: 10.1002/prot.26186 (PMC8671168; doi:10.1002/prot.26186)
Supplement: Supplementary file 1 — Appendix S1: Supporting Information [file PROT-90-58-s001.docx]

**Supplementary Materials**

**Description of distance-guided template-free modeling**

In the distance-guided free modeling, ab initio models are mostly generated from predicted residue-residue distance with a customized trRosetta and our inhouse ab initio predictor - DFOLD (**Figure S1**). DFOLD uses predicted inter-residue distance and secondary structures as restraints to guide 3D structure modeling based on distance geometry and simulated annealing[1]. To predict inter-residue distances, at first, two kinds of MSAs are generated. One is generated by searching a target against the Uniclust30[2], UniRef90[3] and Metaclust[4] databases using DeepAln and DeepMSA. The other one is generated by using HHblits to search against the BFD database[4,5]. Then, a combined MSA is built by combining the two kinds of MSAs and filtering out redundancy according to the sequence identity threshold of 95%. The three kinds of MSAs are used for distance prediction and template-free modeling. For two server predictors - MULTICOM-CONSTRUCT and MULTICOM-CLUSTER, the MSAs are then fed into trRosetta to predict the inter-residue geometries and tertiary structures for the target protein. For another three server predictors - MULTICOM-HYBRID, MULTICOM-DEEP, MULTICOM-DIST, the distance maps predicted by DeepDist from the MSAs are used to substitute the default distance maps predicted by the deep networks in trRosetta for model generation. About 50-100 models are built by trRosetta using different probability thresholds on distance map predictions. Top 10 models selected by the ranking methods (e.g., SBROD) are added into the template-free model pool. In these three servers, additional models generated by DFOLD are also added into the template-free model pool for another round of protein model ranking.


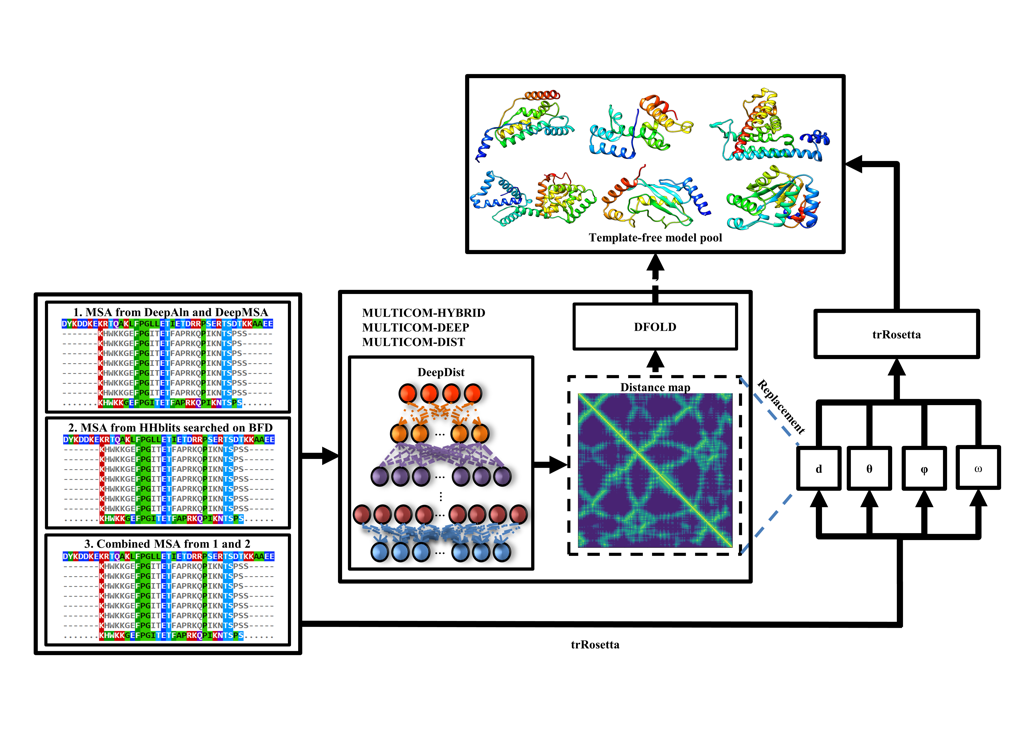


**Figure S1.** MULTICOM distance-based template-free structure predictors in CASP14.

**
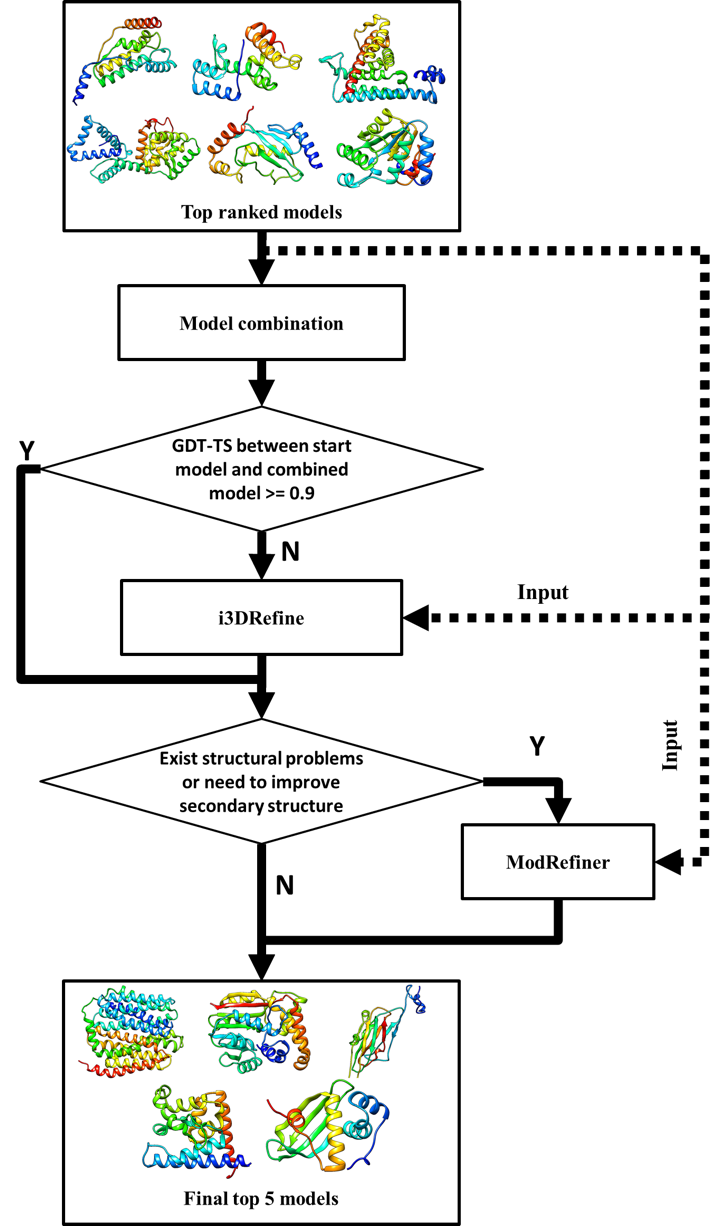
**

**Figure S2**. The standard protocol for model refinement and combination.

**Figure S3.** **(A)** The native structure of target T1034-D1; **(B)** the best model selected by the quality assessment; **(C)** the top1 model predicted by MULTICOM.

**Table S1.** Top 20 CASP14 server predictors on 58 TBM domains evaluated by the assessor’s formula (GDT_HA + (SG + lDDT + CAD) / 3 + ASE) in terms of the sum of Z-scores larger than -2.0, where GDT_HA is GDT High Accuracy score, SG the Sphere Grinder score, lDDT the local Distance Difference Test score, CAD the Contact Area Difference score, and ASE the Accuracy Self Estimate score. Multiple servers from the same group are denoted by the same number in the superscript.

| Predictor Name | Sum Z-score (>-2.0) | Avg Z-score (>-2.0) | Sum Z-score (>0.0) | Avg Z-score (>0.0) |
| --- | --- | --- | --- | --- |
| BAKER-ROSETTASERVER^[1]^ | 38.0718 | 0.6564 | 52.0907 | 0.8981 |
| RaptorX^[2]^ | 26.5604 | 0.4579 | 40.5722 | 0.6995 |
| Zhang-Server^[3]^ | 24.6831 | 0.4256 | 39.018 | 0.6727 |
| QUARK^[3]^ | 23.1323 | 0.3988 | 37.3346 | 0.6437 |
| FEIG-S^[4]^ | 18.8587 | 0.3252 | 35.7677 | 0.6167 |
| Seok-server^[5]^ | 12.1097 | 0.2088 | 29.7808 | 0.5135 |
| Zhang-TBM^[3]^ | 9.3442 | 0.1611 | 31.5684 | 0.5443 |
| Zhang-CEthreader^[3]^ | 7.8989 | 0.1362 | 31.4171 | 0.5417 |
| MULTICOM-DEEP^[6]^ | 4.6145 | 0.0796 | 20.8194 | 0.359 |
| MULTICOM-CONSTRUCT^[6]^ | 3.9167 | 0.0675 | 19.75 | 0.3405 |
| MULTICOM-HYBRID^[6]^ | 1.5593 | 0.0269 | 18.9844 | 0.3273 |
| MULTICOM-CLUSTER^[6]^ | -0.6846 | -0.0118 | 17.1611 | 0.2959 |
| MULTICOM-DIST^[6]^ | -2.0824 | -0.0359 | 18.4336 | 0.3178 |
| Yang-Server^[7]^ | -3.1289 | -0.0539 | 23.9924 | 0.4137 |
| Yang_FM^[7]^ | -3.634 | -0.0627 | 23.11 | 0.3984 |
| Zhang_Ab_Initio^[3]^ | -8.5749 | -0.1478 | 19.034 | 0.3282 |
| IntFOLD6^[8]^ | -8.6664 | -0.1494 | 19.0746 | 0.3289 |
| Yang_TBM^[7]^ | -10.1453 | -0.1749 | 20.5955 | 0.3551 |
| CATHER^[9]^ | -10.4899 | -0.1809 | 21.2685 | 0.3667 |
| BAKER-ROBETTA^[1]^ | -16.3391 | -0.0068 | 18.7091 | 0.3742 |

**Table S2.** Top 20 CASP14 server predictors on 38 TBM/FM or FM domains evaluated by the assessor’s formula (GDT_TS + QCS + 0.1 * Molprobity) in terms of the sum of positive Z-scores, where GDT-TS is the Global Distance Test Score and QCS the Quality Control Score. Multiple servers from the same group are denoted by the same number in the superscript.

| Predictor Name | Sum Z-score (>-2.0) | Avg Z-score (>-2.0) | Sum Z-score (>0.0) | Avg Z-score (>0.0) |
| --- | --- | --- | --- | --- |
| QUARK^[1]^ | 38.5983 | 1.0157 | 41.0331 | 1.0798 |
| Zhang-Server^[1]^ | 37.833 | 0.9956 | 40.2236 | 1.0585 |
| Zhang-CEthreader^[1]^ | 32.8307 | 0.864 | 37.4477 | 0.9855 |
| Zhang-TBM^[1]^ | 30.5569 | 0.8041 | 33.439 | 0.88 |
| Zhang_Ab_Initio^[1]^ | 25.1922 | 0.663 | 29.5266 | 0.777 |
| tFold-CaT^[2]^ | 20.554 | 0.5409 | 24.0149 | 0.632 |
| BAKER-ROSETTASERVER^[3]^ | 20.0555 | 0.5278 | 27.0395 | 0.7116 |
| tFold^[2]^ | 19.2048 | 0.5054 | 23.7377 | 0.6247 |
| tFold-IDT^[2]^ | 19.1705 | 0.5045 | 23.2788 | 0.6126 |
| Yang-Server^[4]^ | 17.9829 | 0.4732 | 22.4134 | 0.5898 |
| Yang_FM^[4]^ | 16.1171 | 0.4241 | 21.3196 | 0.561 |
| MULTICOM-HYBRID^[5]^ | 14.6973 | 0.3868 | 20.1767 | 0.531 |
| FoldX^[6]^ | 14.4935 | 0.3814 | 18.905 | 0.4975 |
| MULTICOM-DIST^[5]^ | 13.8536 | 0.3646 | 19.2372 | 0.5062 |
| Yang_TBM^[4]^ | 13.6895 | 0.3603 | 20.0208 | 0.5269 |
| MULTICOM-DEEP^[5]^ | 13.6875 | 0.3602 | 19.6591 | 0.5173 |
| FALCON-DeepFolder^[7]^ | 13.5072 | 0.3555 | 18.6262 | 0.4902 |
| TOWER^[8]^ | 13.2279 | 0.3481 | 18.1948 | 0.4788 |
| MULTICOM-CONSTRUCT^[5]^ | 12.0938 | 0.3183 | 18.4616 | 0.4858 |
| RaptorX^[9]^ | 11.8695 | 0.3124 | 16.2479 | 0.4276 |

**Table S3.** The results of the model combination on the 23 targets, including GDT-TS scores of the original or combined models, their GDT-TS differences, and the number of models that were used in model combination (bold font denotes a higher score).

| Target | GDT-TS of the original model | GDT-TS of the combined model | Number of models that were used in model combination | GDT-TS difference  (combined – original) |
| --- | --- | --- | --- | --- |
| T1024 | 0.6061 | **0.6112** | 20 | 0.0051 |
| T1026 | **0.6849** | 0.6781 | 3 | -0.0068 |
| T1029 | 0.404 | 0.404 | 20 | 0 |
| T1034 | 0.8237 | **0.8702** | 20 | 0.0465 |
| T1037 | **0.526** | 0.5235 | 2 | -0.0025 |
| T1039 | **0.278** | 0.2764 | 5 | -0.0016 |
| T1040 | 0.2865 | **0.2885** | 2 | 0.002 |
| T1042 | 0.3424 | **0.3451** | 7 | 0.0027 |
| T1043 | 0.1639 | **0.1655** | 2 | 0.0016 |
| T1046s1 | 0.7188 | **0.7569** | 20 | 0.0381 |
| T1055 | 0.7152 | **0.7254** | 16 | 0.0102 |
| T1056 | 0.5917 | **0.5962** | 11 | 0.0045 |
| T1065s1 | 0.8844 | **0.8866** | 20 | 0.0022 |
| T1065s2 | 0.801 | **0.8699** | 20 | 0.0689 |
| T1068 | 0.5293 | **0.5433** | 9 | 0.014 |
| T1074 | 0.5 | **0.5038** | 2 | 0.0038 |
| T1078 | 0.7578 | **0.7674** | 20 | 0.0096 |
| T1083 | 0.8315 | **0.856** | 20 | 0.0245 |
| T1084 | **0.9155** | 0.912 | 20 | -0.0035 |
| T1086 | 0.3937 | **0.397** | 4 | 0.0033 |
| T1089 | 0.6711 | **0.683** | 20 | 0.0119 |
| T1090 | 0.5688 | 0.5688 | 16 | 0 |
| T1099 | 0.5421 | **0.5463** | 2 | 0.0042 |

**Table S4.** The TM-scores of the template-based or template-free models and the E-values of Top 1 template hit on 33 FM or FM/TBM domains (excluding T1052-D3, T1061-D1, T1061-D2, T1080-D1, T1085-D2 due to unavailable true structures for this analysis or lack of predicted full-length template-based or template-free models). The e-value of the top 1 template hit for each target is also reported. The template-free models are more accurate than the template-based models on 32 out of 33 FM or FM/TBM domains (97%) (bold font denotes a higher score).

| Target | E-value of Top 1 Template Hit | TM-score of the template-based modeling | TM-score of the template-free modeling |
| --- | --- | --- | --- |
| T1027-D1 | 5.00E+01 | 0.22 | **0.36** |
| T1029-D1 | 1.30E+02 | 0.21 | **0.46** |
| T1031-D1 | 1.30E+01 | 0.21 | **0.29** |
| T1033-D1 | 1.00E+02 | **0.27** | 0.26 |
| T1035-D1 | 4.40E+00 | 0.2 | **0.78** |
| T1037-D1 | 1.60E+02 | 0.1 | **0.69** |
| T1038-D1 | 4.40E+00 | 0.16 | **0.33** |
| T1038-D2 | 4.40E+00 | 0.18 | **0.61** |
| T1039-D1 | 4.40E-03 | 0.23 | **0.28** |
| T1040-D1 | 2.80E+00 | 0.18 | **0.24** |
| T1041-D1 | 9.60E-01 | 0.15 | **0.62** |
| T1042-D1 | 3.20E+02 | 0.12 | **0.46** |
| T1043-D1 | 1.30E+02 | 0.18 | **0.19** |
| T1046s1-D1 | 8.90E+02 | 0.37 | **0.59** |
| T1047s1-D1 | 2.90E+00 | 0.12 | **0.39** |
| T1047s2-D1 | 2.00E+01 | 0.12 | **0.77** |
| T1047s2-D3 | 2.00E+01 | 0.2 | **0.7** |
| T1049-D1 | 5.90E+02 | 0.23 | **0.67** |
| T1053-D1 | 1.50E+01 | 0.46 | **0.64** |
| T1053-D2 | 1.50E+01 | 0.08 | **0.77** |
| T1055-D1 | 1.80E+00 | 0.22 | **0.68** |
| T1058-D1 | 1.30E+00 | 0.16 | **0.82** |
| T1064-D1 | 3.10E+00 | 0.21 | **0.25** |
| T1065s2-D1 | 1.90E+02 | 0.34 | **0.84** |
| T1070-D1 | 4.10E+01 | 0.19 | **0.35** |
| T1074-D1 | 2.30E+02 | 0.16 | **0.52** |
| T1082-D1 | 1.10E+02 | 0.3 | **0.49** |
| T1090-D1 | 1.70E+02 | 0.41 | **0.57** |
| T1093-D1 | 3.70E-95 | 0.37 | **0.4** |
| T1093-D3 | 3.70E-95 | 0.19 | **0.2** |
| T1094-D2 | 3.20E-53 | 0.25 | **0.28** |
| T1096-D1 | 1.50E+02 | 0.08 | **0.69** |
| T1096-D2 | 1.50E+02 | 0.08 | **0.74** |
| Average |  | 0.211 | 0.513 |

**Table S5.** The TM-scores of the top-1 template-based or template-free models on 47 TBM domains (excluding T1052-D1, T1052-D2, T1061-D3, T1091-D1, T1091-D2, T1091-D3, T1091-D4, T1085-D1, T1085-D3, T1086-D1, T1086-D2 due to unavailable true structures for this analysis or lack of predicted full-length template-based or template-free models). The average TM-score of the template-free models is 0.703, higher than 0.636 of the template-based models. The template-free models are more accurate than the template-based models on 28 out of 47 TBM domains (59.6%) (bold font denotes a higher score).

| Target | E-value of Top 1 Template Hit | TM-score of the template-based modeling | TM-score of the template-free modeling |
| --- | --- | --- | --- |
| T1024-D1 | 2.30E-34 | 0.84 | **0.9** |
| T1024-D2 | 2.30E-34 | 0.81 | **0.82** |
| T1025-D1 | 7.30E-24 | **0.89** | 0.77 |
| T1026-D1 | 3.00E+01 | **0.76** | 0.58 |
| T1028-D1 | 1.30E-27 | **0.82** | 0.74 |
| T1030-D1 | 1.10E-18 | 0.36 | **0.65** |
| T1030-D2 | 1.10E-18 | 0.29 | **0.49** |
| T1032-D1 | 4.80E-13 | 0.56 | **0.62** |
| T1034-D1 | 1.80E+01 | 0.81 | **0.86** |
| T1036s1-D1 | 2.60E-227 | **0.89** | 0.19 |
| T1045s1-D1 | 5.30E-33 | **0.95** | 0.88 |
| T1045s2-D1 | 5.70E-15 | 0.67 | **0.78** |
| T1046s2-D1 | 2.30E+02 | 0.62 | **0.64** |
| T1047s2-D2 | 2.00E+01 | 0.11 | **0.84** |
| T1050-D1 | 5.40E-61 | **0.78** | 0.76 |
| T1050-D2 | 5.40E-61 | 0.82 | **0.88** |
| T1050-D3 | 5.40E-61 | **0.87** | 0.81 |
| T1054-D1 | 1.60E-15 | 0.41 | **0.64** |
| T1056-D1 | 5.40E-23 | **0.57** | 0.56 |
| T1057-D1 | 4.80E-47 | 0.83 | **0.9** |
| T1058-D2 | 1.30E+00 | 0.49 | **0.71** |
| T1060s2-D1 | 9.50E-07 | 0.72 | 0.72 |
| T1060s3-D1 | 6.10E-02 | 0.57 | **0.69** |
| T1065s1-D1 | 5.50E+02 | 0.43 | **0.84** |
| T1067-D1 | 4.20E-25 | 0.48 | **0.54** |
| T1068-D1 | 3.60E-68 | 0.54 | **0.65** |
| T1070-D2 | 4.10E+01 | 0.62 | **0.87** |
| T1070-D3 | 4.10E+01 | 0.21 | **0.61** |
| T1070-D4 | 4.10E+01 | 0.21 | **0.72** |
| T1073-D1 | 5.70E-03 | 0.72 | **0.77** |
| T1076-D1 | 3.70E-66 | **0.94** | 0.92 |
| T1078-D1 | 8.50E-05 | 0.51 | **0.83** |
| T1079-D1 | 5.50E-96 | 0.75 | **0.83** |
| T1083-D1 | 5.20E-02 | 0.51 | **0.82** |
| T1084-D1 | 2.50E-02 | 0.75 | **0.86** |
| T1087-D1 | 6.50E-01 | **0.67** | 0.42 |
| T1089-D1 | 1.10E-31 | **0.81** | 0.78 |
| T1092-D1 | 2.80E-80 | **0.59** | 0.58 |
| T1092-D2 | 2.80E-80 | **0.79** | 0.68 |
| T1093-D2 | 3.70E-95 | **0.64** | 0.5 |
| T1094-D1 | 3.20E-53 | **0.59** | 0.47 |
| T1095-D1 | 2.50E-97 | **0.64** | 0.51 |
| T1099-D1 | 7.30E-65 | **0.54** | 0.51 |
| T1100-D1 | 8.80E-20 | 0.44 | **0.67** |
| T1100-D2 | 8.80E-20 | 0.54 | **0.64** |
| T1101-D1 | 1.30E-23 | 0.7 | **0.84** |
| T1101-D2 | 1.30E-23 | **0.82** | 0.74 |
| Average |  | 0.636 | 0.703 |

**References:**

1. Wu T, Guo Z, Hou J, et al. DeepDist: real-value inter-residue distance prediction with deep residual network. *BMC Bioinformatics,* 2020.

2. Mirdita M, von den Driesch L, Galiez C, et al. Uniclust databases of clustered and deeply annotated protein sequences and alignments. *Nucleic acids research* 2017;45:D170-D176.

3. Consortium U. UniProt: a worldwide hub of protein knowledge. *Nucleic acids research* 2019;47:D506-D515.

4. Steinegger M, Söding J. Clustering huge protein sequence sets in linear time. *Nature communications* 2018;9:1-8.

5. Steinegger M, Mirdita M, Söding J. Protein-level assembly increases protein sequence recovery from metagenomic samples manyfold. *Nature methods* 2019;16:603-606.
